# Death of Dr. Thomas

**Published:** 1897-11

**Authors:** 


Death of Dr. Thomas.—Dr. Jerome E. Thomas, of Mexia, Texas, died at his home in that city, September 18th, 1897. He was born in Alabama May 1st, 1852, and came with his parents to Texas from Louisiana, where they had lived up to 187C, and settled in Leon county. He attended a course of lectures at Tulane University, New Orleans, in 1874, and began practicing medicine. He attended the Missouri Medical College in St. Louis in the winter of 1875-6, and graduated from that institution in the spring of 1886. He then located at Brewer, Freestone county, where he established himself in practice. He was married at Mexia, previous to graduating, to Miss Anna E. Curtis, of Mexia. In 1881, Dr. Thomas removed to Mexia, where he enjoyed a large practice up to the date of his death. In the spring of 1887, Dr. Thomas took a post-graduate course at the Louisville Medical College. He was a member of and deacon in the Baptist church, a Royal Arch Mason, and a High Priest in that order at the time of his death. Dr. Thomas leaves a wife and four children, and, we are pleased to learn, they are well provided for, the doctor having kept his life well insured.

Dr. Thomas was a man of strong individuality. He had many warm friends, and few enemies. He was a member in first-class standing both in the State and District Medical Association, and was a member of the District Board of Medical Examiners. He was a staunch friend and a longtime supporter of the TEXAS MEDICAL JOURNAL, and the JOURNAL joins in the universal regret of his too early death. He was just forty-five years of age.

